# Paraganglioma of the filum terminale: case report

**DOI:** 10.1186/1477-7819-7-95

**Published:** 2009-12-11

**Authors:** Alessandro Landi, Roberto Tarantino, Nicola Marotta, Pierluigi Rocco, Manila Antonelli, Maurizio Salvati, Roberto Delfini

**Affiliations:** 1 Department of Neurosurgery, University of Rome Sapienza, Rome, Italy; 2 Department of Pathological Anatomy, University of Rome Sapienza, Rome, Italy

## Abstract

**Background:**

Paragangliomas affecting the filum terminale are extremely rare, benign tumors. The literature yielded thirty-two cases of paraganglioma in this site.

**Case presentation:**

A 49 year-old-man, whose presenting symptoms were low back pain and left leg weakness, was diagnosed as having a paraganglioma of the filum terminale. The clinical, histological and radiological characteristics of this case, that brings the total number of cases described to 33, are discussed in the light of published data.

**Conclusions:**

This extremely rare pathology can usually be successfully treated by total surgical resection, which represents the gold standard. In the event of incomplete removal, assiduous long-term follow-up is mandatory.

## Introduction

Paragangliomas affecting the filum terminale are extremely rare, benign tumours and only 32 cases have been reported in the international literature to date: their characteristics are summarized in Table 1. In the present study, another case of paraganglioma of filum terminale is described. The patient was a man aged 49 whose initial symptoms consisted of low back pain, radicular pain and left leg weakness. The clinical onset, histopathological features and radiological appearance of this pathological entity are discussed in the light of published data.

**Table 1:** Review of the literature from 2007 to 2009.

AUTHORS	AGE AND SEX	SYMPHTOMS	DURATION	TREATMENT	RECURRENCE
Igren et Al	58 F	LBP + sciatica	17 yrs	GTR	no

Sonneland	32 M	LBP	7 mos	GTR	no

	62 M	LBP + sciatica	6 wks	GTR	no

	52 M	LBP	3 yrs	STR + RXT	recurrence after 9 yrs

	57 M	LBP + sciatica	7 yrs	STR	no

	67 F	LBP + sciatica	1 yr	GTR	no

	49 M	LBP + sciatica	6 yrs	GTR + RXT	no

	61 M	LBP + sciatica	14 mos	GTR	no

	47 F	LBP + sciatica	1 yrs	STR + RXT	recurrence after 1 yr

	50 M	LBP + sciatica	3 wks	GTR	no

	71 F	LBP + sciatica	7 yrs	GTR	no

	56 M	paraparesys	1 yrs	GTR	no

	52 F	LBP + sciatica	3 mos	GTR + RXT	no

	53 M	LBP + sciatica	2 yrs	GTR + RXT	no

	48 F	LBP + sciatica	3 yrs	GTR	no

	36 M	LBP	NS	GTR	no

	39 M	LBP	15 yrs	GTR	no

	48 M	LBP + sciatica	1,5 yrs	GTR	no

	40 F	LBP + sciatica	2 yrs	GTR	no

	50 F	LBP + sciatica	15 yrs	GTR	no

	59 M	LBP + sciatica	2 yrs	GTR	no

	58 F	LBP + sciatica	7 mos	GTR	no

	66 F	LBP + sciatica	many yrs	GTR	no

	62 F	LBP + sciatica	many mos	GTR	no

	39 F	LBP + sciatica	10 yrs	GTR + RXT	no

	30 F	LBP + sciatica	4,5 yrs	GTR	no

	69 F	claudicatio	1 yrs	GTR	no

Russel et Al	61 F	LBP	many yrs	GTR	no

	56 F	progressive paraplegia	10 days	GTR	no

Moran et Al	22 F	LBP	NS	GTR	no

	44 M	LBP	NS	GTR	no

Sousa et Al	51 M	LBP + sciatica	3 yrs	GTR	no

Landi et Al	49 M	sciatica + weakness at lower left limb	3 mos	GTR	no

Demographic aspects of previous 32 cases of paraganglioma of filum terminale, addicted with the our case.

## Case description

**Figure 1:**
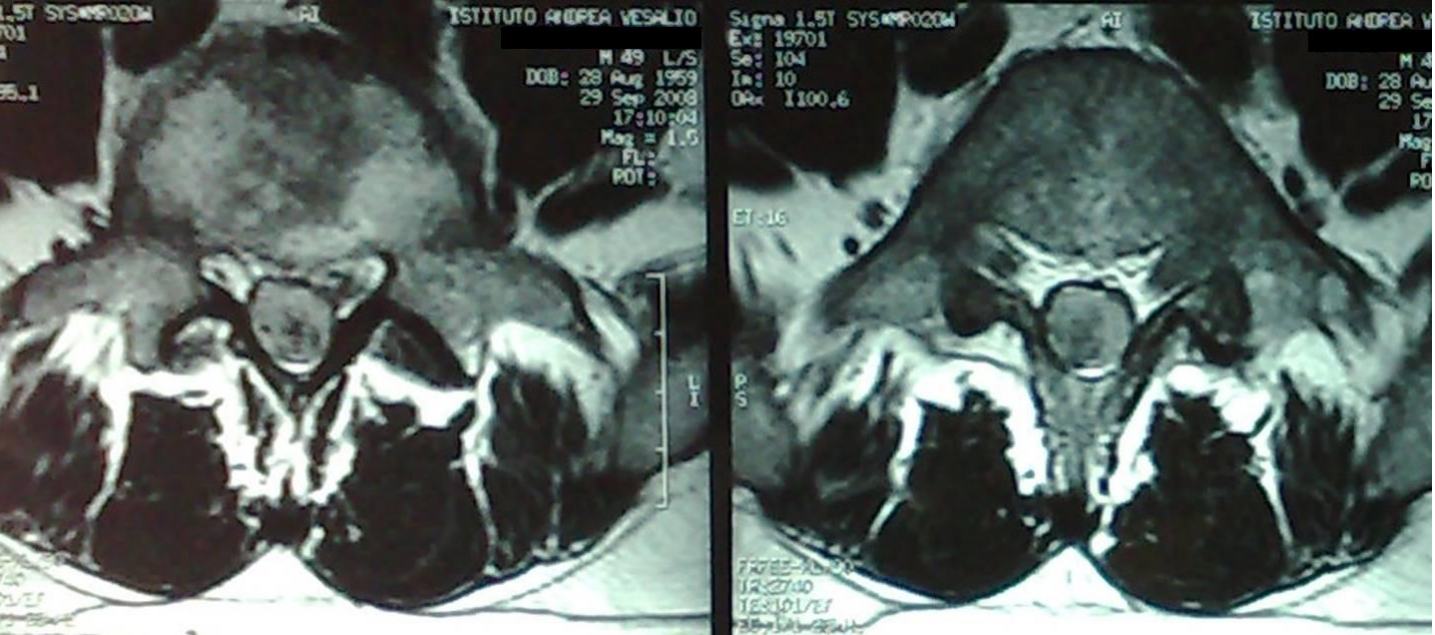
**Preoperative MRI**. Axial T1 weighted images with Gd-DTPA showed an enhancing well defined lesion at L4-L5 level

**Figure 2:**
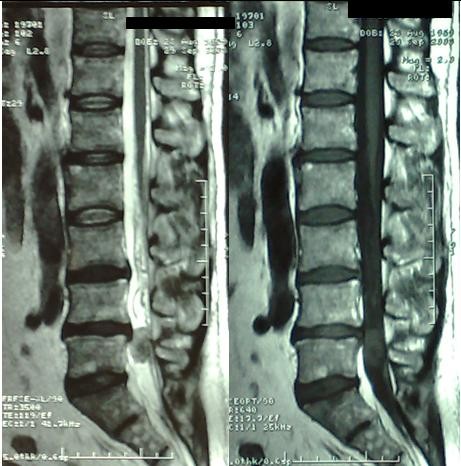
**Preoperative MRI**. Sagittal T2 and T1 weighted images with Gd-DTPA.

**Figure 3:**
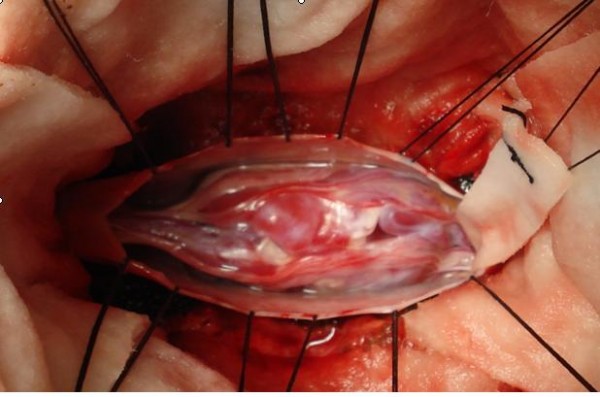
**Intraoperative picture of the lesion, after durotomy**.

**Figure 4:**
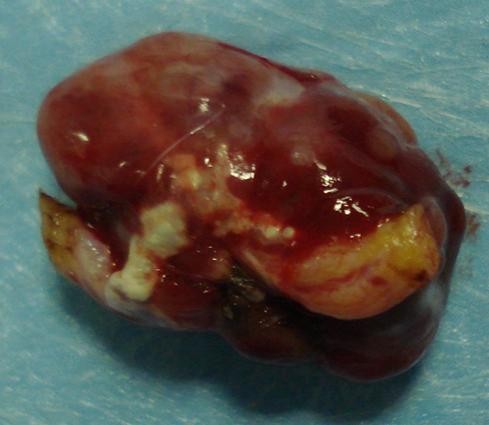
**Macroscopic aspect of the lesion after the en-block removal**.

**Figure 5:**
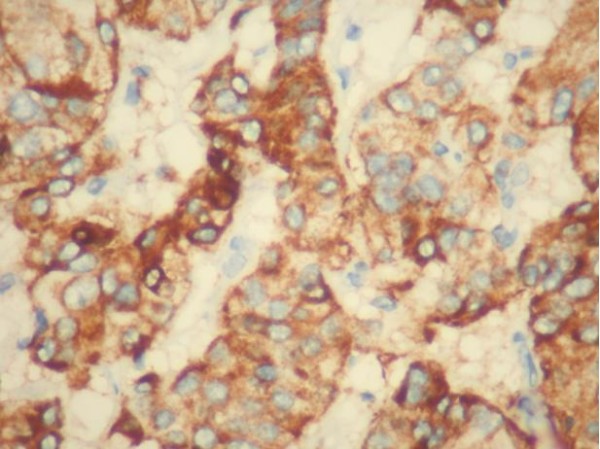
**Postoperative MRI: sagittal T1, T2 and T1 with Gd-DTPA scans that showed the complete removal of the lesion**.

**Figure 6:**
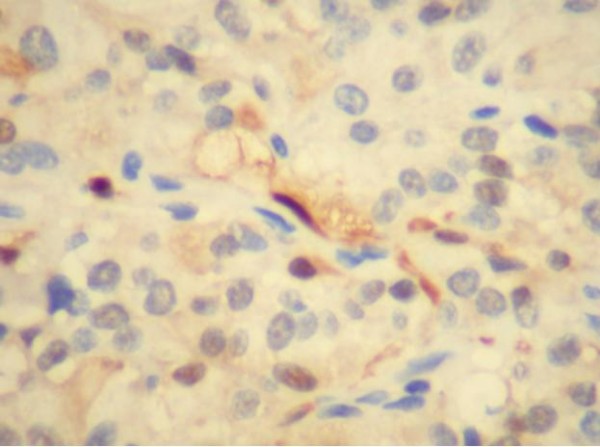
**Postoperative MRI: axial T1, T2 and T1 with Gd-DTPA scans**.

**Figure 7:**
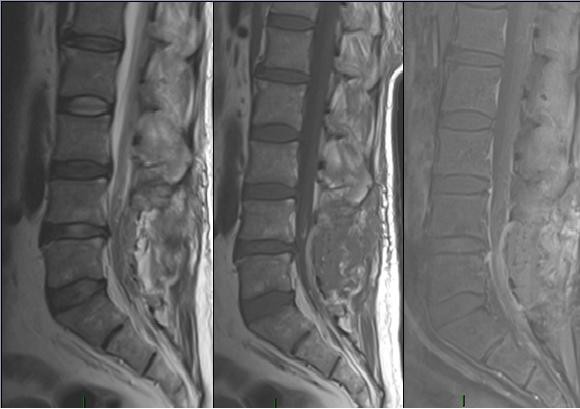
**Sustentacular cells immunoreaction for S-100 protein**.

**Figure 8:**
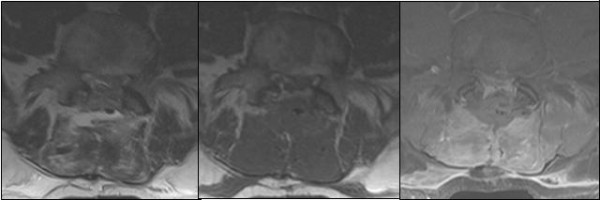
**Immunohistochemical reactivity of chromogranine**.

This 49 year-old man was admitted with a 3-month history of increasing left leg weakness accompanied by progressive low back pain that radiated to the left leg. Painful symptoms were worsened by movement, coughing and upper body rotation. Sphincter dysfunction was absent. Neurological evaluation revealed tenderness in the region of the paravertebral muscles: a straight raise test of the left leg produced posterior thigh pain at 30°. Moderate weakness of the left leg was also observed and dorsal flexion of the foot in the area innervated by L5, revealed a 1/5 reduction of strength. Pinprick sensation was diminished but no dermatomal distribution was identified. Tendon reflexes, peri-anal sensitivity and anal tone were normal. Magnetic resonance imaging of the lumbosacral spinal segment, before and after Gadolinium administration, disclosed an L4-L5 oval shaped lesion measuring 3.3 × 2 × 1 cm. The lesion was intradural and extramedullary and was surrounded by bands of low signal intensity on T1 and T2-weighted images [fig.1, fig.2]; it presented marked contrast enhancement after gadolinium administration. There were no signs or symptoms indicating catecholamine hyper-secretion, such as hypertension, psycho-motor distress or headache. Therefore, in the light of the published data [1], blood laboratory tests to measure the levels of dopamine, epinephrine, noradrenaline and vanillymandelic acid were not deemed necessary. A standard L4-L5 laminectomy was performed to gain access to the intradural lesion of the filum terminale. Was then removed "en bloc" via a standard durotomy [Fig. 3]. The lesion had the appearance of an irregular-shaped nodule of the dimension previously indicated by MRI [fig.4]. it had an elastic consistency and was well-encapsulated and vascularised, adhering to the filum terminale but not to any other structures. During "en bloc" removal, the filum was resected cranially to the lesion, bluntly dissecting it from the caudal roots which appeared displaced but showed no evidence of infiltration. The patient made an excellent postoperative recovery. The pain and weakness in his left leg regressed immediately and completely and by the second day after surgery ha was able to walk without aid. Gadolinium-enhanced MRI of the lumbosacral region confirm total excision of the lesion and no evidence of pathological enhancement was observed [fig.5, fig 6]. on the sixth postoperative day the patient was discharged. Histological evaluation of the tumour showed it to be made up of uniform cells arranged in large lobules, or smaller nests, known as "Zellballen". The aggregated chief cells were surrounded by capillaries, present throughout the lesion. There was a flattened layer of sustentacular cells, positive for S100 [fig 7] which encompassed both the lobules and the "Zellballen". Immunohistochemical testing gave a positive reaction for chromogranin in the chief cells [fig. 8].

## Discussion

Paraganglioma is a neuro-endocrine tumour, it had a neuroectodermal origin and derives from the embryonic sympathetic and parasympathetic nervous system. The first authors to describe this pathological entity were Miller and Torack in 1970, denominating it a secretory ependymoma [2], whereas Lerman was the first to coin the term paraganglioma of the cauda equina in 1972 [3]. The great majority of paragangliomas (85-90%) arise in the adrenal gland whereas extra-adrenal tumours are principally situated in the jugular glomus of the carotid body [4]. Paragangliomas with an extra-spinal localization may be multiple and have a higher incidence in subjects with a positive family history since an association with Von Hippel Lindau disease and MEN II has been observed. In the central nervous system, the most common extra spinal localizations of paraganglioma are the petrous ridge, pineal gland and sella turcica [5-8]. The spinal localization is uncommon, with an incidence in the general population calculated at 0.07 per 100.000 inhabitants. Tumours affecting the carotid body and glomus region are usually parasympathetic whereas the spinal variety are typically sympathetic. In the spine, these tumours most commonly occur in the cauda equina and filum terminale region, with a slight male prevalence. They are believed to arise from sympathetic neurons which originate in the lateral horn of the spinal cord and follow the course of the nerve roots [1,9,10]. Another possible locus of tumour origin consists of the heterotopic neurons that lie along the branches proximal to the sympathetic trunk. In the lumbar spine the origin of these lesions could be the paraganglia situated in the cauda equina. However, since it has been suggested [11] that some components of the diffuse neuro-endocrine system are the product of a local differentiation and do not derive from neural crest tissue, the possibility that ependymal cells play a role in the development of these lesions cannot be ruled out. The cauda equina localization of paraganglioma has been well-documented. In the filum terminale, however, this tumour is much rarer and English-language databases have yielded only 32 cases to date [1,12,13]. Biologically, these lesions are generally benign. A genetic basis has been suggested on the strength of observations of familiarity and an association with leiomyosarcoma, chordoma or pituitary adenoma. Regarding their onset, the mean patient age is 46 years and clinical presentation usually consist of low back pain, sometimes associated with radicular pain. A cauda equina syndrome is unusual, despite the fact that the tumour takes up the whole diameter of the spinal canal. Sensory and motor deficits are also uncommon. Incontinence (urine and faeces) is relatively rare [14,15]. The features of the case we observed were concordant with the published data in that the patient had a 3-month history of worsening low back pain and radicular pain with a moderate left leg weakness but no sphincter dysfunction. The role of MRI in the diagnosis and treatment of this condition is paramount, owing to the abundance of anatomical information it provides, although a correct diagnosis may pose some problems. On T1-weighted images, the lesion usually has an isointense appearance, whereas it is hyperintense on T2-weighted sequences with enhancement after Gadolinium administration. These features are common to other intradural lesions, such as schwannomas, mixopapillary ependymomas, ependymomas, meningiomas, metastatic tumours and dermoid tumours or lipomas [14,16]. In the diagnostic work-up of paraganglioma, two observations may be helpful for differentiation: 1) a defect in the serpiginous structure between the conus and the lesion, suggesting dilatation of the serpentine vessels, which is uncommon in schwannomas and ependymomas. 2) a hypointense appearance of the tumour rim on T2-weighted sequences, suggesting para-magnetic effects caused by hemosiderin, which is typical of vascular tumours since hemosiderin discolouration is a sign of prior hemorrhage (3). Currently, the functional imaging method preferred for localizing extra-adrenal or adrenal paragangliomas is Scintigraphy with I-MIBG. Although there can be no doubt of the high level of sensitivity and specificity of this method, it also has some drawbacks. These consist of relatively high levels of irradiaton exposure and failure to identify non-secreting paragangliomas, in other words the majority of those situated in the cauda equina and filum terminale [1]. Surgical treatment aims at completely excision of the lesion that is usually well-encapsulated, as in the case we observed. Histopathological diagnosis is based on the characteristics typical of paraganglioma, namely an organoid or "Zellballen" organization of argyrophilic cells which are circumscribed by vascular stroma, immunohistochemical testing with a positive reaction for chromogranine and sustentacular cell immune reaction for S100 protein [9]. In the international Classification of Disease for Oncology (ICD-O), paragangliomas of the cauda equina and filum terminale are classified as "tumours of neuro-epithelial tissue" in the sub-group of "Neuronal and Mixed Neuronal-Glial Tumours" (8680/1 morphology code): In the WHO grading system they are classified as grade 1 [1,17]. The prognosis of these lesions is good, as long as they are completely removed. In fact, subtotal excision is not infrequently followed by local recurrence and even a distant metastatic spread has been described [10]. Consequently, prognosis is better in lesions that are well-encapsulated. When complete removal is not feasible, radiotherapy may be considered, although resistance to this form of treatment has been documented [9]. As far as chemotherapy is concerned, at the time being it does not appear to play a role in the management of these lesions. The median free interval between surgical removal and local recurrence is estimated to be approximately 6 years [18]. However, the fact that isolated cases of recurrence have been observed as many as 20 years after surgical treatment [18] indicates that an assiduous long-term follow-up is mandatory whenever complete removal has not been achieved.

## Conclusions

Paraganglioma of the filum terminale is an extremely uncommon condition. It usually presents with low back pain and radicular pain and may be accompanied by a loss of strength in some cases. Magnetic resonance with Gadolinium administration gives useful information for surgical planning but is not diagnostic. The goal of surgical treatment is total resection of the lesion that guarantees cure in the majority of cases. Patients in whom complete removal is not feasible require assiduous long-term follow-up.

## Consent statement

Written informed consent was obtained from the patient for publication of this case report and any accompanying images. A copy of the written consent is available for review by the Editor-in-Chief of this journal

## Competing interests

The authors have not been influenced by any financial or personal relationship with people or organizations in preparation of this study

## Authors' contributions

All authors have made substantial contributions to in the design of the article: AL was responsible for editing, English editing, correction, search of the literature, conception and design, and has contributed in surgical technique. RT was responsible for editorship of the manuscript and has contributed in surgical technique. NM was responsible for the search of the literature. PR was responsible for the search of the literature. MS was responsible for the oncology consulting. MA was responsible for the histology consulting and pathology examination. RD is the principal surgeon and was responsible for editing.
